# Supplementary material for: Investigation of Outbreaks of Extended-Spectrum Beta-Lactamase-Producing Klebsiella Pneumoniae in Three Neonatal Intensive Care Units Using Whole Genome Sequencing
Source: Antibiotics (Basel). 2020 Oct 16;9(10):705. doi: 10.3390/antibiotics9100705 (PMC7650633; doi:10.3390/antibiotics9100705)
Supplement: Supplementary file 1 [file antibiotics-09-00705-s001.zip › Table S2.docx]

**Table S1**. Genetic characteristics of genomes found in outbreaks of ESBL-producing *K. pneumoniae* in three neonatal intensive care units. Grey marked numbering indicates the number of found genes while a blank box indicates absence.

| Sample number |  | Capsule | | Siderophores | | Fluoroquinolones | | Tetracyclines | | | Aminoglycosides | | | | | | | Sulfonamides | | Chloramphenicol | | Beta-lactamases | | | | | | | | | | | |
| --- | --- | --- | --- | --- | --- | --- | --- | --- | --- | --- | --- | --- | --- | --- | --- | --- | --- | --- | --- | --- | --- | --- | --- | --- | --- | --- | --- | --- | --- | --- | --- | --- | --- |
|  | ST | K-locus | O-antigen type | Yersiniabactin | ICEKp | QnrB1 | QnrS1 | TetA | TetB | TetD | StrA | StrB | aph(3')-Iia | aac(6')-Ib- | aac(3)-IIa | aac(3)-IId | aadA2 | sul1 | sul2 | CatA2 | catB3 | LAP-2 | OXA-1* | TEM-1 | SHV-1 | SHV-11 | SHV-27 | SHV-28 | SHV-33 | SHV-38 | SHV-187 | CTX-M-14 | CTX-M-15 |
| 1 | ST985# | KL39 | O1v2 | 16 | 12 | 1 |  | 1 |  |  | 1 | 1 |  | 1 | 1 |  |  |  | 1 |  | 1 |  | 1 | 1 |  |  |  |  |  |  | 1 |  | 1 |
| 4 | ST985# | KL39 | O1v2 | 16 | 12 | 1 |  | 1 |  |  | 1 | 1 |  | 1 | 1 |  |  |  | 1 |  | 1 |  | 1 | 1 |  |  |  |  |  |  | 1 |  | 1 |
| 5 | ST464 | KL17 | O1V1 |  |  |  | 1 |  |  |  | 1 | 1 | 1 |  |  |  |  |  | 1 |  |  |  |  |  |  |  |  |  |  | 1 |  |  | 1 |
| 7 | ST985# | KL39 | O1v2 | 16 | 12 | 1 |  | 1 |  |  | 1 | 1 |  | 1 | 1 |  |  |  | 1 |  | 1 |  | 1 | 1 |  |  |  |  |  |  | 1 |  | 1 |
| 8 | ST13 | KL3 | O1v2 |  |  | 1 |  | 1 |  |  | 1 | 1 |  | 1 | 1 |  |  |  | 1 |  |  |  | 1 |  | 1 |  |  |  |  |  |  |  | 1 |
| 9 | ST464 | KL17 | O1V1 |  |  |  | 1 |  |  |  | 1 | 1 | 1 |  |  |  |  |  | 1 |  |  |  |  |  |  |  |  |  |  | 1 |  |  | 1 |
| 10 | ST985# | KL39 | O1v2 | 16 | 12 | 1 |  | 1 |  |  | 1 | 1 |  | 1 | 1 |  |  |  | 1 |  | 1 |  | 1 | 1 |  |  |  |  |  |  | 1 |  | 1 |
| 11 | ST46 | KL64 | O1V1 |  |  | 1 |  | 1 |  |  | 1 | 1 |  | 1 |  |  |  |  | 1 |  | 1 |  | 1 | 1 |  |  | 1 |  |  |  |  |  | 1 |
| 12 | ST37# | KL15 | O4 | 5 | 6 |  | 1 |  |  |  | 1 | 1 |  |  |  |  | 1 | 1 | 1 |  |  |  |  |  |  | 1 |  |  |  |  |  |  | 1 |
| 13 | ST35 | KL110 | O1V1 |  |  |  | 1 | 1 |  |  |  |  |  |  |  | 1 |  | 1 |  |  |  | 1 |  |  |  |  |  |  | 1 |  |  | 1 |  |
| 14 | ST37# | KL15 | O4 | 5 | 6 |  | 1 |  |  |  | 1 | 1 |  |  |  |  | 1 | 2 | 1 | 1 |  |  |  |  |  | 1 |  |  |  |  |  |  | 1 |
| 15 | ST37# | KL15 | O4 | 5 | 6 |  | 1 |  |  |  | 1 | 1 |  |  |  |  | 1 | 2 | 1 | 1 |  |  |  |  |  | 1 |  |  |  |  |  |  | 1 |
| 16 | ST37# | KL15 | O4 | 5 | 6 |  | 1 |  |  |  | 1 | 1 |  |  |  |  | 1 | 2 | 1 | 1 |  |  |  |  |  | 1 |  |  |  |  |  |  | 1 |
| 17 | ST37# | KL15 | O4 | 5 | 6 |  | 1 |  |  |  | 1 | 1 |  |  |  |  | 1 | 2 | 1 | 1 |  |  |  |  |  | 1 |  |  |  |  |  |  | 1 |
| 25 | ST105 | KL102 | O1V1 |  |  |  |  |  | 1 |  | 1 | 1 |  |  |  |  |  |  | 1 |  |  |  |  | 1 | 1 |  |  |  |  |  |  |  | 1 |
| 30 | ST35# | KL110 | O1V1 |  |  |  | 1 | 1 |  |  |  |  |  |  |  | 1 |  | 1 |  |  |  | 1 |  |  |  |  |  |  | 1 |  |  | 1 |  |
| 31 | ST35# | KL110 | O1V1 |  |  |  | 1 | 1 |  |  |  |  |  |  |  | 1 |  | 1 |  |  |  | 1 |  |  |  |  |  |  | 1 |  |  | 1 |  |
| 38 | ST268 | KL20 | O2V1 | 10 | 4 |  |  | 1 |  |  | 1 | 1 | 1 |  |  |  | 1 | 1 | 1 |  |  |  |  |  |  | 1 |  |  |  |  |  |  | 1 |
| 40 | ST15 | KL57 | O1V2 |  |  |  |  |  |  | 1 | 1 | 1 | 1 |  |  |  |  | 1 |  |  |  |  |  | 1 |  |  |  | 1 |  |  |  |  | 1 |

* Identified using raw Fastq data and dual sequencing technology assembly but failed to assemble with only Illumina reads. #dominant outbreak clonal group.
