# Supplementary figures and images for: Investigation of Outbreaks of Extended-Spectrum Beta-Lactamase-Producing Klebsiella Pneumoniae in Three Neonatal Intensive Care Units Using Whole Genome Sequencing
Source: Antibiotics (Basel). 2020 Oct 16;9(10):705. doi: 10.3390/antibiotics9100705 (PMC7650633; doi:10.3390/antibiotics9100705)

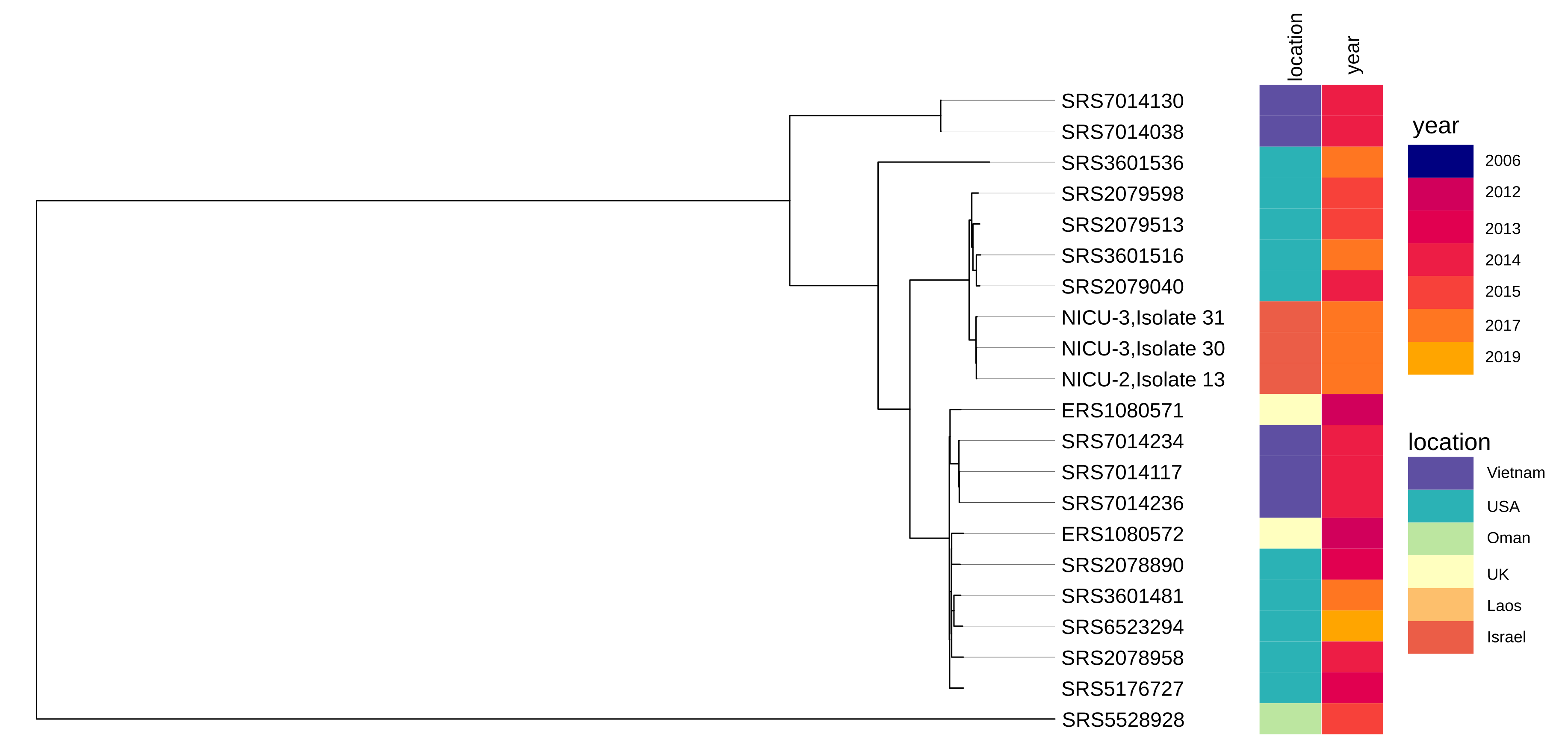

Supplement: Supplementary file 1 [file antibiotics-09-00705-s001.zip › Fig. S1.tif]

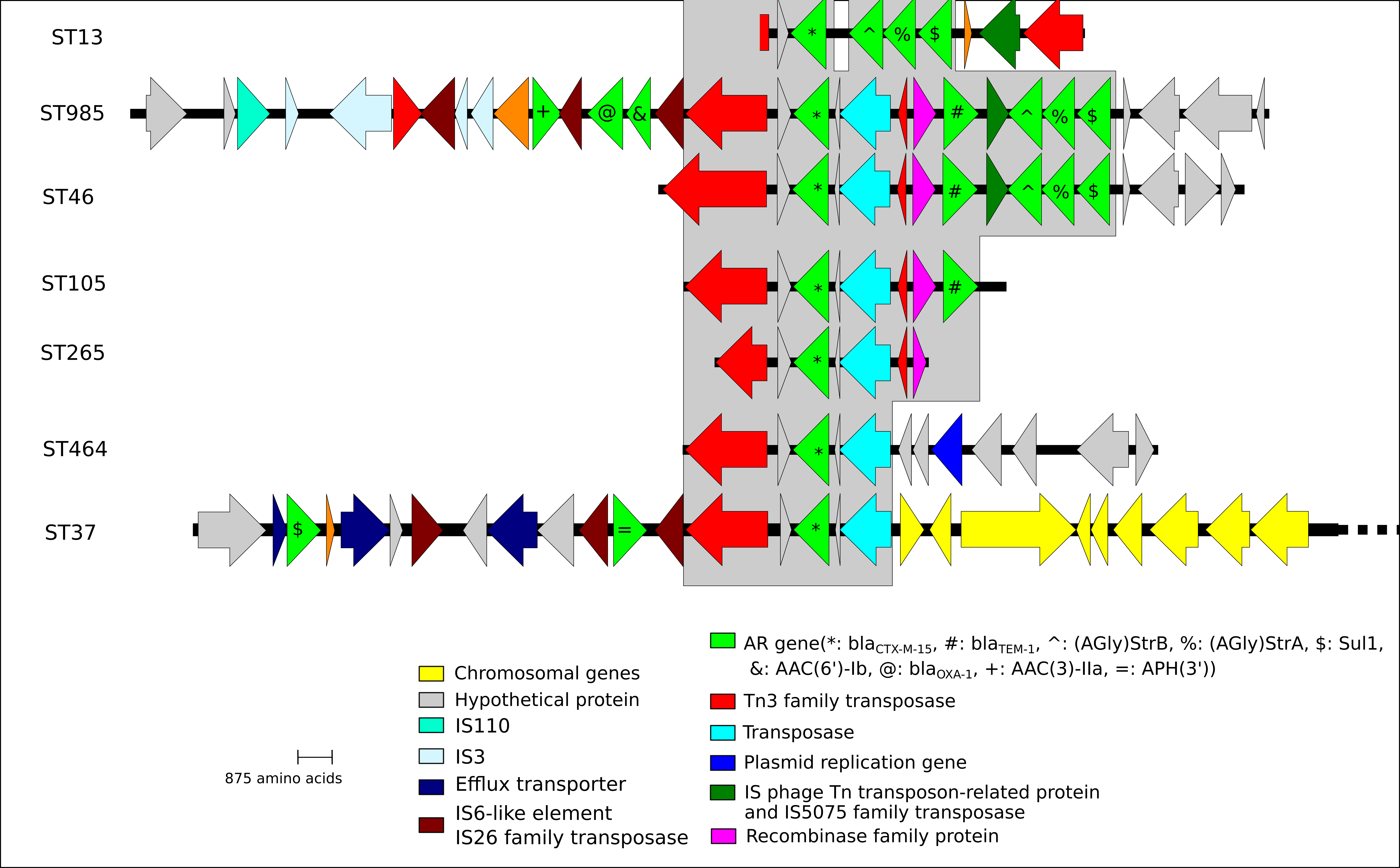

Supplement: Supplementary file 1 [file antibiotics-09-00705-s001.zip › Fig. S3.png]

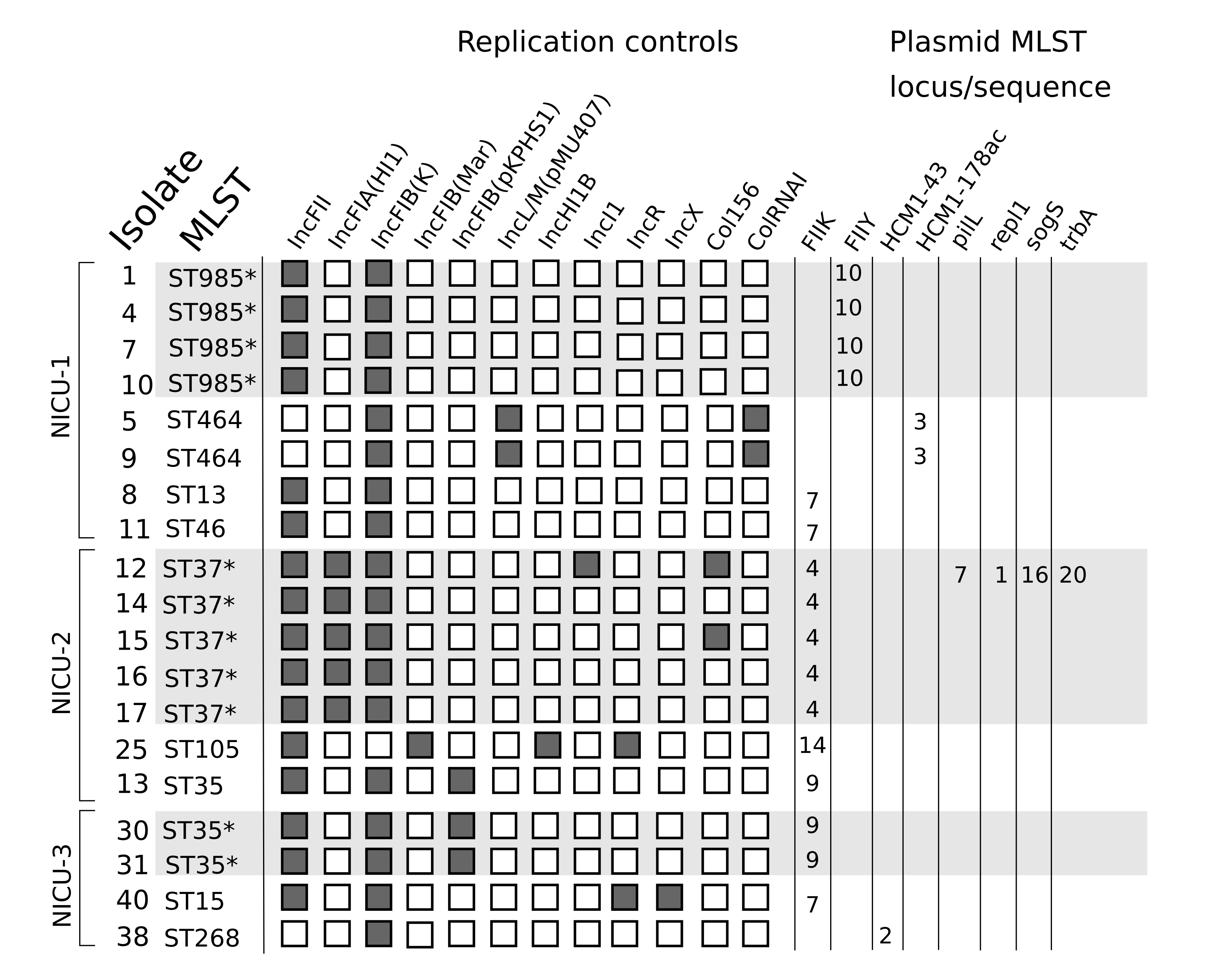

Supplement: Supplementary file 1 [file antibiotics-09-00705-s001.zip › Figure S2.tif]
